# Simulation-based what-if analysis for controlling the spread of Covid-19 in universities

**DOI:** 10.1371/journal.pone.0246323

**Published:** 2021-02-01

**Authors:** Navid Ghaffarzadegan

**Affiliations:** Department of Industrial and Systems Engineering, Virginia Tech, Falls Church, Virginia, United States of America; Harvard Medical School, UNITED STATES

## Abstract

A simulation model is developed to analyze the spread of covid-19 in universities. The model can be used to conduct a what-if analysis and estimate infection cases under different policies. For proof-of-concept, the model is simulated for a hypothetical university of 25,000 students and 3,000 faculty/staff in a U.S. college town. Simulation results show that early outbreaks are very likely, and there is no silver bullet to avoid them. Instead, a combination of policies should be carefully implemented. The results suggest (almost) full remote university operations from the beginning of the semester. In a less-preferred alternative, if universities decide to have students attend in person, they should encourage remote operations for high-risk individuals, conduct frequent rapid tests, enforce mask use, communicate with students and employees about the risks, and promote social distancing. Universities should be willing to move to remote operations if cases rise. Under this scenario, and considering implementation challenges, many universities are still likely to experience an early outbreak, and the likelihood of having a case of death is worrisome. In the long run, students and faculty react to the risks, and even if universities decide to continue operations, classes are likely to have very low in-person attendance. Overall, our analysis depicts several sources of system complexities, negative unintended consequences of relying on a single policy, non-linear incremental effects, and positive synergies of implementing multiple policies. A simulation platform for a what-if analysis is offered so marginal effectiveness of different policies and different decision-making thresholds for closure can be tested for universities of varying populations.

## 1. Introduction

During a pandemic, universities face major challenges in operating on-campus actives. Such was the case during the Fall of 2020 when the world was coping with the covid-19 pandemic. Despite the risks, many U.S. universities decided to hold in-person classes, at least partially, and invited students to campus [1, 2]. In-person classes are often justified based on the need for quality instruction and engagement in university environments [3]. The risks, however, are clear, as it is likely that student arrivals lead to outbreaks on university campuses. What can universities do to decrease the likelihood of an on-campus outbreak? In the case of covid-19, several policies have been suggested to prevent a potential outbreak [4, 5]. However, since this was the first experience of its kind in almost a century, limited data existed to evaluate policy alternatives for university settings. This study, which was mainly conducted prior to the fall 2020 semester, and in response to the risk of covid-19 pandemic in universities, uses a model-based simulation approach to investigate a wide range of scenarios.

Model-based approaches for analyzing the spread of infectious diseases have been widely used, and many of the past models are applicable in this context. The conventional SEIR (Susceptible, Exposed, Infected, and Removed) models provide a framework that is relatively easy to implement and simulate [6], and several examples have already been applied to study the spread of covid-19 in contexts other than universities [7–10]. However, the university context, especially in a U.S. college town, has specific characteristics that justify developing models specific to universities. At the beginning of a semester, a considerable population of students, often totaling more than the town’s residents, suddenly arrive (in a very short time period) to a small town that is experiencing no or low numbers of disease cases. Some of the arriving students have already been exposed to the disease and are unaware of it. Their arrival can change the dynamics of the disease spread throughout the university and the town. Furthermore, universities by design promote social interaction, and students are very likely to gather and become involved in activities that lead to a sudden growth in case numbers. These activities are not necessarily limited to classes. Arguably, social interactions are among the most important factors that make the early years of the college experience joyful. Students are also more likely to patronize restaurants and public transportation that can further increase the likelihood of infection and transmission. In addition, social gatherings outside of the campus cannot by controlled by the university administration. Altogether, universities face a dilemma: on one hand, they feel the need to keep their campus operating; on the other hand, they are responsible for the health of their students and employees.

In this paper, we develop a simulation platform to analyze the effects of different policies on the spread of the disease and the likelihood of student or employee death in a typical university in a U.S. college town. To that end, we build a dynamic model of a pandemic for a hypothetical college that includes behavioral and policy responses that often play a substantial role in healthcare settings [11]. The model is generic and can be applied to different universities. In our proof-of-concept test, we parametrize the model for a hypothetical case wherein 25,000 students arrive on campus in September and interact with a population of 3,000 faculty/staff, and the semester is planned to comprise a 90-day period. The model is simulated to estimate infection cases over the semester and under different policies of social distancing, testing, risk communication, remote operation, and mask use enforcement. In addition to analyzing the pattern, our intention is to offer a web-based platform that could be used for a what-if analysis. Such platforms are essential for experiential learning [12, 13]. While the main analysis of this paper was done prior to the fall 2020 semester, we include a post-analysis reflection in which we examine the model’s fidelity in replicating the data from our home institution.

## 2. Modeling

A wide range of pandemic models have been put forth to study the spread of covid-19 in different contexts [7–9]. Our simulation model in this study follows the same thread of infectious disease compartmental modeling and includes behavioral and administrative reactions to changes in the number of cases. Most conventional models have assumed constant parameters to represent social interactions, which is a major limitation in projecting future trends (two exceptions are references 9 and 10).

**Fig 1:**
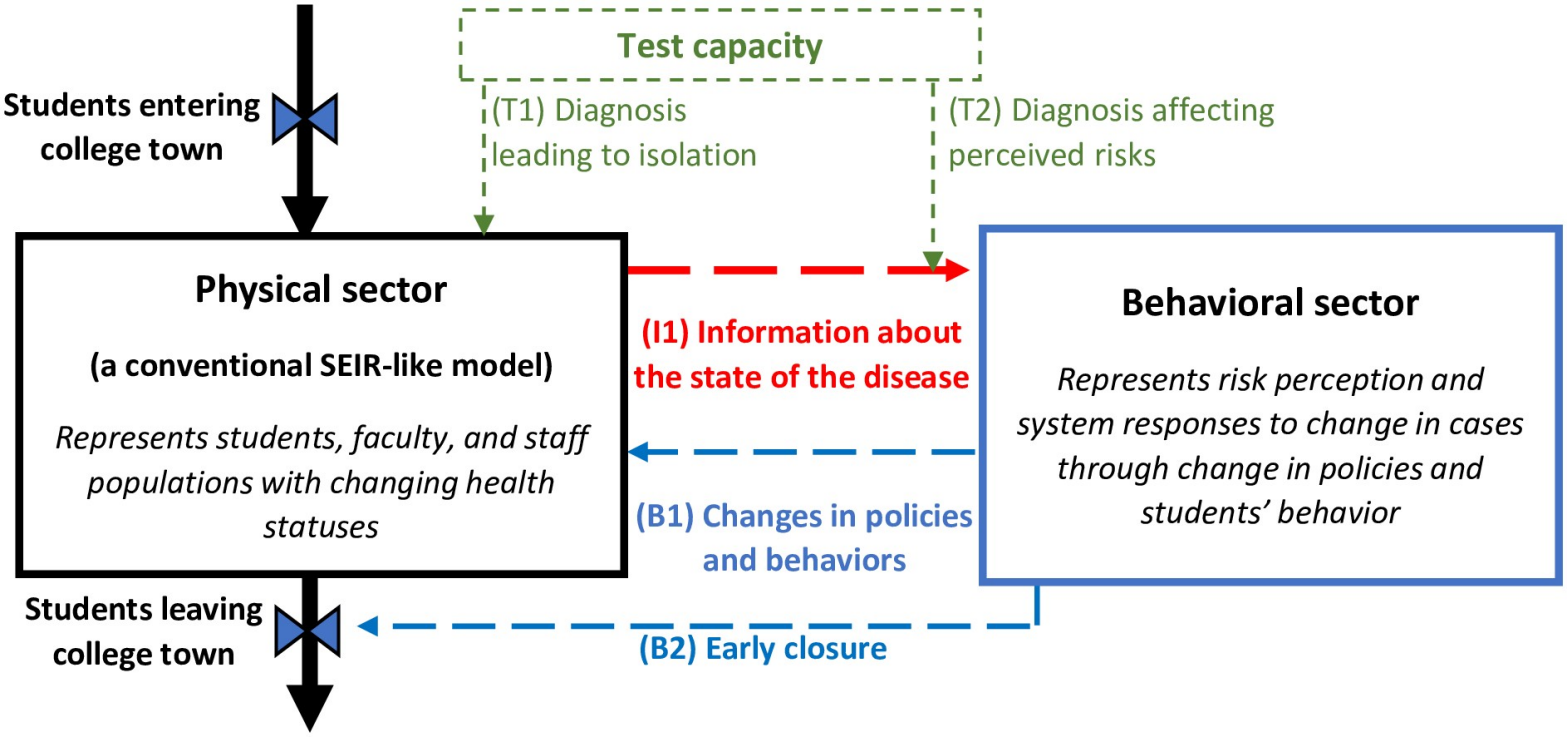
A conceptual representation of the entire model.

A conceptual, high-level, representation of our model is depicted in Fig 1. On the physical side, an SEIR-like model that accounts for test coverage and its effects on decreasing contacts through isolation is developed. On the behavioral side, potential changes in institutional and personal policies are modeled. These policies include early school closure and a decline in personal contacts that feed back to the physical sector affecting the spread of the virus. In our model we assume that students arrive early in the semester (in the figure, the inflow to the physical sector) and do not leave unless the university is closed (the outflow from the physical sector). School closure can happen at the end of the semester or as a response to the increasing number of cases. In reality, some students may decide to leave the college early on or commute to their family home, but those are rare cases and strongly discouraged by the institutions.

In particular, we include testing in our model and contrast cases based on documented vs. undocumented cases. This approach is consistent with how Li et al. separated cases [7], but contrasts with many other approaches that differentiate cases based on the level of symptoms [14]. Test frequency can affect both sectors of the model the following ways. First it can affect the physical sector by increasing the fraction of diagnosed cases, leading to more isolation (link T1). Second, with more tests, more cases are diagnosed, thereby affecting perceived risks of the administration and students (link T2) and influencing decisions and behaviors that feed back the rate at which the disease spreads (links B1 and B2).

The model includes several feedback mechanisms that can affect the final outcomes. Inside the physical sector, transmission dynamics are among the most important mechanisms (not shown in Fig 1), representing the fact that more active cases of infection results in more new cases. The sector also includes the potential saturation of susceptibles, which becomes important as time passes and the susceptibles’ number declines leading to herd immunity. The other two important mechanisms relate to interactions between the behavioral sector and transmission dynamics. As covid cases increase, more will be discovered through tests, which can influence risk perceptions of the students and university administration (link I1). Based on decision-making models and sensitivity to the unfolding cases, at least one of these two scenarios are likely: a change in students’ behavior by practicing more social distancing and avoiding risky behavior (link B1) or an early school closure (link B2). The relative strength of links B1 and B2 can be different in different institutions.

We consider potential interactions between students and employees who often live in nearby areas. This helps understand the spread of the disease among such population of college town residents. In reality, other forms of interaction include various daily interactions in grocery stores, restaurants, and public transportation, which we do not explicitly represent in the model. A more accurate representation of social interactions will require a detailed model that includes social network structures and contact heterogeneities. In the next sections, we formulate these sectors. All model variables and parameters are listed in Table 1 for quick reference. In the following section, we document the model structure and parameter values following the format suggested by Jalali and colleagues [15].

**Table 1:** Notations of model variables and parameters.

Notation	Definition
*a*_*x*_	fraction of total arrival rate with status x. x is *S*, *E I*_*U*_, or *R*_*U*_.
*A*	daily arrival rate of students. *A*_*x*_ arrival rate with status x. x is *S*, *E I*_*U*_, or *R*_*U*._
*B*_*x*_	population in the age category of x. x is younger than 30, between 30 and 60, and over 60.
*C*	average contact rate
*C*_*max*_	constant contact rate absent any exogenous or endogenous changes in social behavior
*e*_*m*,*i*_	effect of mask adoption
*e*_*Temp*,*i*_	effect of temperature
*E*	exposed: sick, early stage
$\tilde{E}$	daily rate of testing exposed
*F*	Infection fatality rate. *f*_*x*:_ Infection fatality rate for different age categories of x.
*FP*	false positives
*H*	college sensitivity to number of cases
*I*	infection probability given contact
*i*_*N*_	infection probability in the absence of mask in average September temperature
*I*_*D*_	infected documented: sick, late stage, and diagnosed
*I*_*U*_	infected undocumented: sick, late stage, and undiagnosed
*k*_*E*_	normal fraction of testing exposed
*k*_*S*_	normal frequency of testing healthy
*L*_*x*_	leaving rate of students with status *x*. *x* is *S*, *E*, *I*_*U*_, *I*_*D*_, *R*_*U*_, *R*_*D*_ or *FP*.
*N*	total population
*P*	positive cases
$\tilde{p}$	14-day moving average of positive cases
*R*_*D*_	removed documented: diagnosed individuals who recover or die.
*R*_*U*_	removed undocumented: undiagnosed individuals who recover or die.
*R*	all infected in the university (documented or undocumented) who are removed.
*S*	susceptibles: never sick before
$\tilde{S}$	daily rate of testing susceptibles
*T*	average time to evacuate the college town after school closure
*T*_*C*_	test capacity
*T*_*sp*_	test specificity
*T*_*sn*_	test sensitivity
*W*_*N*_	endogenous element of contact rate
*α*	relative infectivity of documented to undocumented cases
*θ*	represents reactive (*θ* = 1) vs. proactive testing (*θ* > 1)
*μ*	relative infectivity of exposed to undocumented cases
*ρ*	test coverage fraction
*τ*_1_	exposure period: average time to move from early to late stage of the disease.
*τ*_2_	infection post-exposure period.
*τ*_*Arrival*_	arrival duration
*φ*	University closure decision, 0: open, 1: closed.

### 2.1. Physical sector

**Fig 2:**
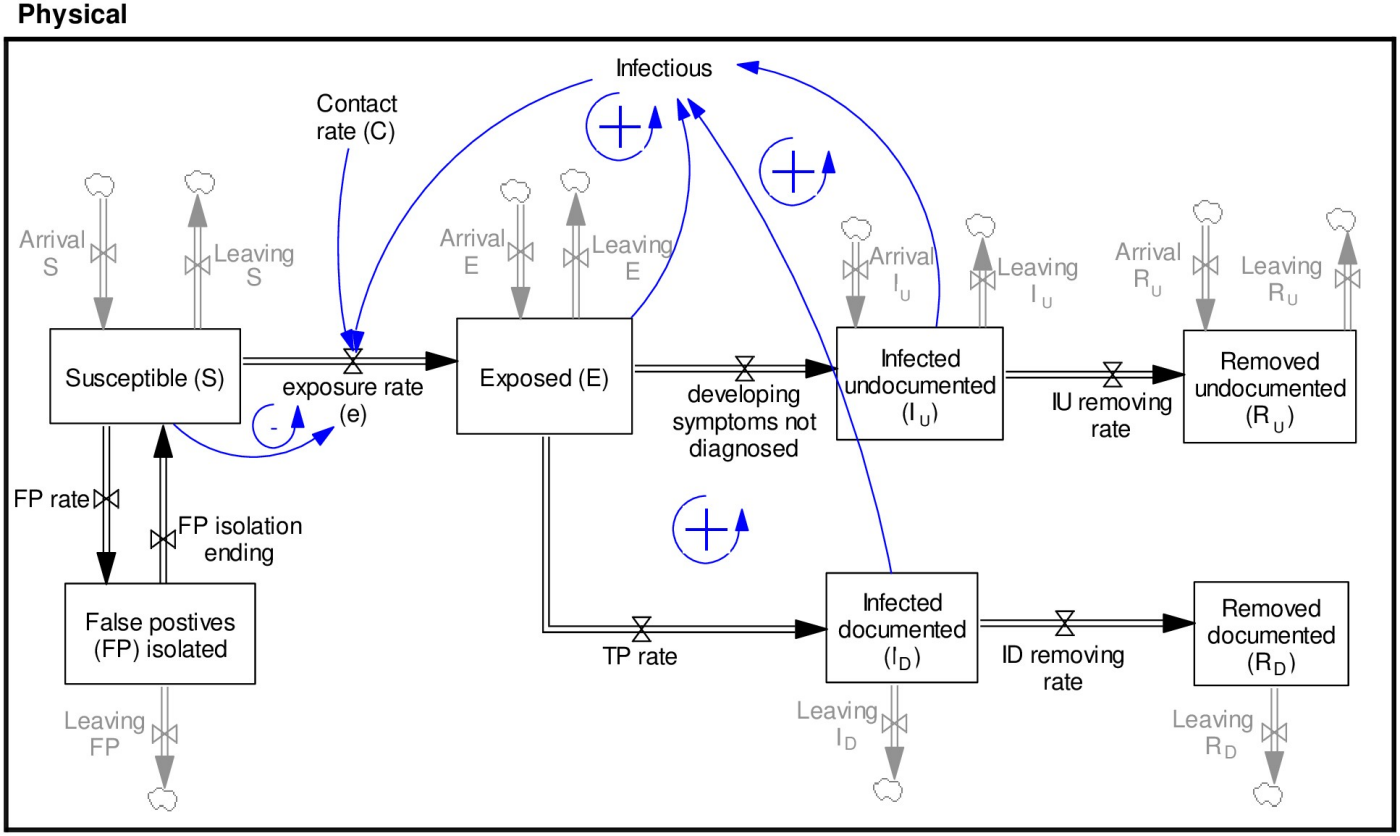
A simple representation of health states and transmission dynamics in the physical sector of the model.

We use a detailed representation of the flow of individuals across different system compartments, as shown in Fig 2. From the left, susceptibles (*S*) move to the exposed state (*E*), staying in this state for an average period of *τ*_1_ days. This is the period in which patients do not show any major symptoms. After the exposure period, a fraction will be diagnosed (infected documented, *I*_*D*_), and the rest will be missed (infected undocumented, *I*_*U*_). The reason for differentiating the groups is that many cases remain asymptomatic [16, 17] or undocumented due to test coverage limits [7]. In addition, test accuracy is limited [18], and it is likely that some infected cases are missed. Furthermore, the infectivity level of each group is different [7]. Each occupant of these groups of documented and undocumented infected individuals will be eventually removed (*R*_*D*_ and *R*_*U*_) on average in *τ*_2_ days. It is assumed that recovery will result in immunity.

In addition, a fraction of susceptible individuals may be wrongly diagnosed and move to false positives (*FP*). We consider that they will potentially self-isolate for an average period of *τ*_2_ days before returning to the susceptible group.

#### 2.1.1. Student arrival and leaving rate

Let *A* be the daily arrival of students and *τ*_*Arrival*_ be the duration of arrival starting from t = 0. At the beginning of the semester, some of the arriving students will possibly be infected presumably with no or mild symptoms. We estimate arrival rate to each compartment of our model. Let *A*_*x*_ represent the arrival rate to the compartment *x* where *x* = {*S*, *E*, *I*_*U*_, *I*_*D*_, *R*_*U*_, *R*_*D*_}. Since we simulate cases identified and documented in the university, $A_{R_{D}}\;=\;A_{I_{D}}\;=\;0$. Let represent the fraction susceptible, exposed, and undocumented infected by *a*_*s*_, *a*_*E*_, and $a_{I_{U}}$, respectively (Eq 1a–1d).

$$A_{S}\;=\;a_{S}A$$ (1a)

$$A_{E}\;=\;a_{E}A$$ (1b)

$$A_{I_{U}}\;=\;a_{I_{U}}A$$ (1c)

$$A_{R_{U}}\;=\;(1-a_{S}-a_{E}-a_{I_{U}})A$$ (1d)

Outflows to the outside of the system boundary represent students’ leaving rate after the semester ends or after early closure. Let *L*_*x*_ be the leaving rate of students where *x* = {*S*, *E*, *I*_*U*_, *I*_*D*_, *R*_*U*_, *R*_*D*_,*FP*} represents student population of different health states. Eq 2 formulates the leaving rate, where *φ* = {0,1} represents university closure decision (*φ* = 1 when university is closed) and *T* is average time to leave the university:

$$L_{x}\;=\;\varphi \frac{x}{T}.$$ (2)

#### 2.1.2. Transmission dynamics

Fig 2 includes transmission feedback loops. The population that transmits the disease includes a fraction of exposed and infected individuals still in contact with susceptibles and has not isolated themselves. We set the relative infectivity of each group by parameters *μ* (relative infectivity of exposed to undocumented cases), and *α* (relative infectivity of documented to undocumented cases). Therefore, (*μE + αI*_*D*_ + *I*_*U*_) is the effective population that transmits the disease:

$$\frac{dS}{dt}\;=\;-iC\frac{S}{N}(\mu E+\alpha I_{D}+I_{U})-[(1-T_{sp})\tilde{S}-\frac{FP}{\tau_{2}}]+A_{S}-L_{S}$$ (3)

$$\frac{dE}{dt}\;=\;iC\frac{S}{N}(\mu E+\alpha I_{D}+I_{U}\text{)}-\frac{E}{\tau_{1}}+A_{E}-L_{E}$$ (4)

$$\frac{dFP}{dt}\;=\;[(1-T_{sp})\tilde{S}-\frac{FP}{\tau_{2}}]-L_{FP},$$ (5)

where *C* is the average contact rate, *i* is the infection probability given contact, and *N* is the total population. As stated, we consider the fact that some susceptibles might be wrongly diagnosed and isolated (False Positives, *FP*). $\tilde{S}$ is the daily rate of testing susceptibles, and *T*_*sp*_ is test specificity. The term $\left[(1-T_{sp})\tilde{S}-\frac{FP}{\tau_{2}}\right]$ represents the outflow from and inflow to False Positives who will be unnecessarily isolated (Eq 5).

The exit rate from exposed (*E*) is equal to $\frac{E}{\tau_{1}}$. A fraction of this group will be tested ($\tilde{E}$) and some will be diagnosed and join *I*_*D*_ and the rest will move to *I*_*U*_ (Eqs 6 and 7):

$$\frac{dI_{D}}{dt}\;=\;T_{sn}\tilde{E}-\frac{I_{D}}{\tau_{2}}-L_{I_{D}}$$ (6)

$$\frac{dI_{U}}{dt}\;=\;(\frac{E}{\tau_{1}}-T_{sn}\tilde{E})-\frac{I_{U}}{\tau_{2}}+A_{I_{U}}-L_{I_{U}},$$ (7)

where *T*_*sn*_ is test sensitivity. For removing rates, we can write:

$$\frac{dR_{D}}{dt}\;=\;\frac{I_{D}}{\tau_{2}}-L_{R_{D}}$$ (8)

$$\frac{dR_{U}}{dt}\;=\;\frac{I_{U}}{\tau_{2}}+A_{R_{U}}-L_{R_{U}}.$$ (9)

#### 2.1.3. Testing

Let *k*_*s*_ be the average frequency of testing susceptibles and *k*_*E*_ the demand fraction among the exposed. Thus, the total demand for testing will be $k_{S}S+k_{E}\frac{E}{\tau_{1}}$. We assume that if total demand is less than the daily test capacity of *T*_*c*_, all demand will be met. If it is more, the limited test capacity will be proportionally distributed between each group based on test coverage fraction of *ρ* (Eqs 10–12):

$$\tilde{E}\;=\;\rho k_{E}\frac{E}{\tau_{1}}$$ (10)

$$\tilde{S}\;=\;\rho k_{S}S$$ (11)

$$\rho =\text{Min}(1,T_{C}/(\theta (k_{S}S+k_{E}\frac{E}{\tau_{1}}))).$$ (12)

In Eq 12, the parameter *θ* is a testing frequency multiplier and represents reactive (*θ* = 1) vs. proactive testing (*θ* > 1). By proactive testing, we simply mean a higher frequency and coverage of testing beyond the normal demand. Thus, higher frequency testing scenarios will be examined by changing *θ*. Higher test frequency can lead to finding more asymptomatic or mild cases.

Finally, the total daily confirmed cases (positive cases, *p*) will be the sum of the daily true positives and false positives:

$$p\;=\;T_{sn}\tilde{E}+(1-T_{sp})\tilde{S}.$$ (13)

#### 2.1.4. Infection probability given contact

We assume that the infection probability after contact with infected (*i*) declines if people use a mask [19], and it slightly increases as the temperature declines [20]. Eq 14 represents the relation:

$$i\;=\;e_{M,i}∙e_{Temp,i}∙i_{N},$$ (14)

where *i*_*N*_ is the normal value of the probability in the absence of a mask in September, *e*_*M*,*i*_(*M*) is the effect of mask adoption (*M*: percent adoption), and *e*_*Temp*,*i*_(*t*) is the exogenous effect of a temperature change. Simple linear functional forms are used for *e*_*M*,*i*_ and *e*_*Temp*,*i*_. We estimate *e*_*M*,*i*_ as 1 − 0.5*M*, where M is the mask adoption fraction. The function provides values consistent with the reference [19] especially in high adoption ranges. We estimate *e*_*Temp*,*i*_ based on Xu and colleagues’ study [20] for the period of September to December at *e*_*Temp*,*I*_ = 1 + 0.0025*t*.

#### 2.1.5. Age categories and faculty and staff risk

Faculty and staff are on average older and have a higher risk of mortality if infected [21]. Age-wise, we assume there are three groups: younger than 30, between 30 and 60, and over 60, represented by *B*_<30_, *B*_30−60_ and *B*_>60_. We assume all individuals of younger generation are arriving from other towns, that is, *B*_*<*30_ = *A*. The last two groups of *B*_30−60_ and *B*_>60_ are faculty and staff, and we assume they live in the area. These groups may decide to work remotely if possible, thus only a fraction of them, represented by *β*_30−60_ and *β*_>60_, will be active and susceptible to the disease. Therefore, after student arrival, the total active population will be *B*_<30_ + *β*_30−60_*B*_30−60_ + *β*_>60_*B*_>60_.

To simplify, we estimate the SEIR categories of each group based on their proportional representation in the population.

For each age group, the corresponding infection fatality rates are represented by *f*_<30_, *f*_30−60_, and *f*_>60_.

#### 2.1.6. Estimation of death probability

In a university context we expect that one case of death would be perceived as a catastrophic outcome that could result in early closure. However, the majority of the population is young, and the likelihood of death for each individual is low. The likelihood of having at least one death will rise if the number of infected increases. Thus, we calculate the probability of at least one case of death throughout the semester.

Let *f* be the probability of death for each individual. The probability of an infected individual surviving *is (*1 − *f*), and the probability of all R number of sick people surviving is (1 − *f*)^*R*^. Thus, the probability of at least one death equals one minus the probability of everyone surviving as shown in Eq 15:

$$probability(death\geq 1)\;=\;1-{\left(1-f\right)}^{R},$$ (15)

where *R* is the cumulative cases removed from the beginning of simulation. Furthermore, in any given time of *t*_*c*_, if no death incident has occurred, we can update the probabilities of future periods by simply replacing *R* with *R* − *R*(*t*_*c*_).

To estimate the death probabilities for different age groups, *R* and *f* is replaced by specific values of that age group.

### 2.2. Behavioral sector

**Fig 3:**
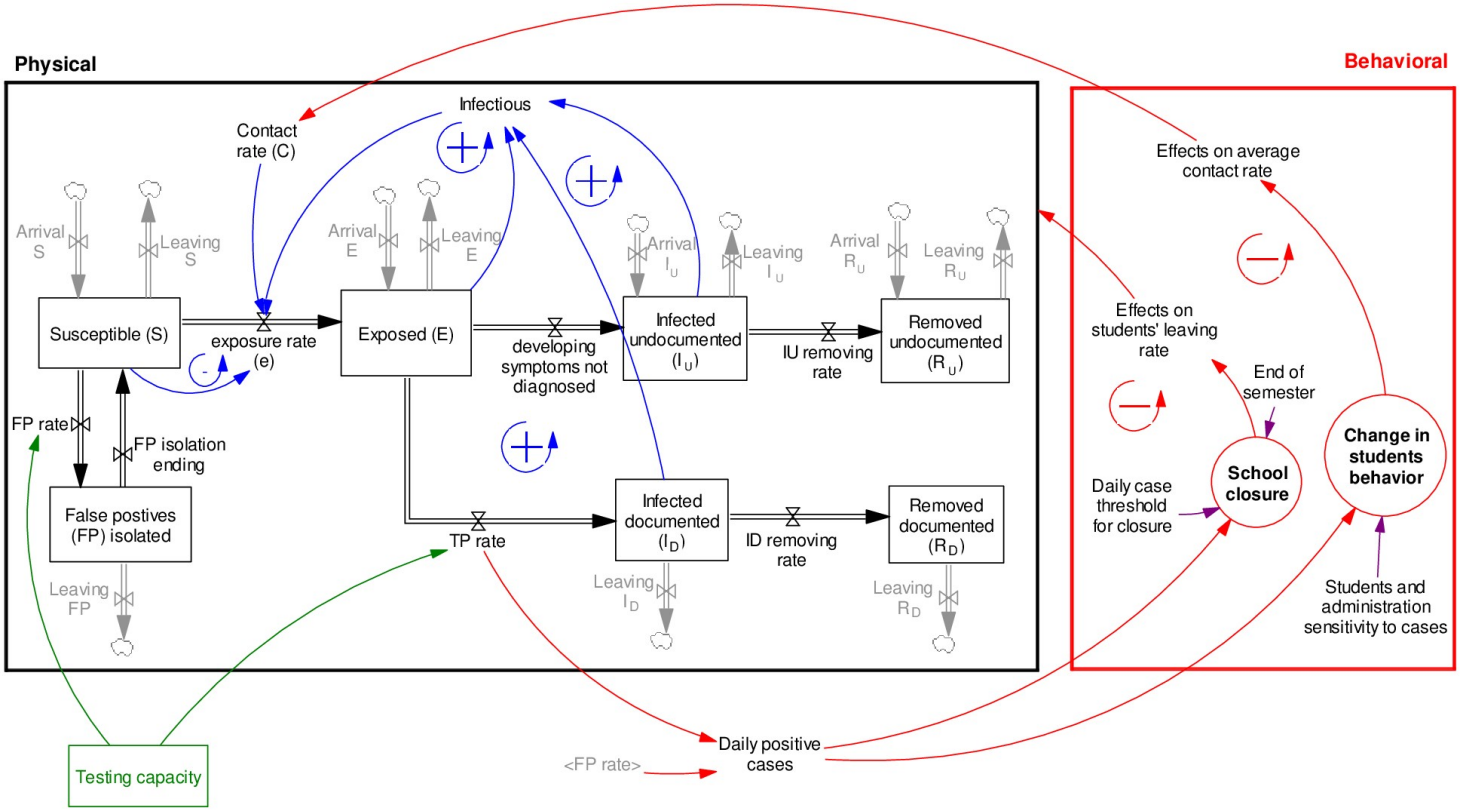
A simple representation of the interaction between the model’s behavioral and physical sectors.

Two major possible mechanisms are included in the model to represent institutional and individual reactions to growing cases. Fig 3 shows the mechanisms, which we discuss in the following section.

#### 2.2.1. University closure and student leaving rate

In a general condition, universities could close sooner than the end of the semester if daily cases pass the administration threshold for closure (see Fig 3). In worst-case scenarios, a case of death is likely to result in early closure as well. In the base run simulation, we assume university closure, *φ* = 1, happens at the end of the semester (*t* > 90) or when the 14-day moving average of daily cases ($\tilde{p}$) reaches 100 cases. This threshold is ad hoc, and we let users change it in our dashboard.

#### 2.2.2. Contact rate and social distancing

For the contact rate (*C*), we write:

$$C\;=\;C_{max}W_{N},$$ (16)

where *C*_*max*_ represents a constant term, that is the average contact rate absent any endogenous changes in social behavior. It is likely that there is a delay in peoples’ noting of daily cases. We consider that more daily cases affect social behavior due to a higher level of perceived risks. *W*_*N*_ represents such an endogenous element of contact rate. This can represent students’ direct reaction to the rise in cases (such as avoiding parties due to the fear of becoming infected), or their reaction due to restrictive policies implemented by the university (such as the fear of suspension if they attend parties). We formulate *W*_*N*_ as a function of the moving average of daily cases ($\tilde{p}$):

$$W_{N}\;=\;exp(-h(\frac{\tilde{p}}{N})).$$ (17)

In this equation, *h* is a coefficient representing the sensitivity of a college to the number of daily cases.

### 2.3. Parameter values

The introduced model is generic and can be adopted for different university settings. To demonstrate its application, we use parameter values based on the most recent covid-19 literature. However, the values mostly come from contexts that could differ from a college town or a university, as data for university contexts are limited. For parameters related to institutional settings (such as the age distribution), we use estimates from our home institution. Table 2 presents the parameter values and their sources.

**Table 2:** Parameter values.

Parameters		Value	Unit	Source
**Disease-transmission**	*τ*_1_	6	day	[21]
	*τ*_2_	10	day	[21, 22].
	*μ*	0.66	scaler	Based on CDC fraction infectivity of exposed to symptomatic is 0.5, and symptomatic to asymptomatic is at 1.33 [21].
	*α*	0.2y	scaler	Relative infectivity of symptomatic. to asymptomatic. is 1.33 [21]. If diagnosed and isolated *y* days after onset, given ~6-day infectivity period, *α* is 1.33(y/6). With proactive testing, some asymp. cases will be diagnosed and the ratio may get closer to 1(y/6). For base run, we assume 0.2y.
	*T*	14	days	Information from home institution.
	*C*_*max*_	13	Person /day	The values of *C*_*max*_ and *i*_*N*_ can’t be independently determined. The product of *C*_*max*_ · *i*_*N*_ is important and it is set in a way that *R*_*0*_ of ~3 is produced. Students are likely to have more contacts and higher *R*_*0*_, thus our estimation is conservative.
	*i*_N_	0.037	Person /day	
**Test-related**	*T*_*sp*_	0.998	scaler	[23]
	*T*_*sn*_	0.8	scaler	[23]
	*T*_*c*_	500	test/ day	For base run, based on data from home institution.
	*k*_*s*_	0.01	1/(person * day)	Assumption of one-time test during the semester per healthy person under the reactive mode of testing.
	*k*_*E*_	0.6	1/ person	Based on fraction of asymptomatic cases of 0.4 [21]. Absent proactive testing, 60% of exposed will be tested if capacity is adequate.
**Population**	*A*	1786	Person /day	*Aτ*_*Arrival*_ = 25,000, number of students in Virginia Tech.
	*τ*_*Arrival*_	14	days	
	*a*_*s*_	0.97	scaler	Estimation based on the disease prevalence in the Commonwealth of Virginia: active cases in August are about 14K, estimated total infected 23K (9K asymptomatic), and 23K pre-symptomatic. Assumed 2.5% are already removed.
	*A*_*E*_	0.003	scaler	
	$a_{I_{U}}$	0.0015	scaler	
	*B*_30−60_	2500	person	Approximately set based on data from home institution.
	*B*_>60_	500	person	
**Policy**	*M*	0	scaler	For base run, it is set 0. For policy test changes to 1.
	*θ*	1	scaler	For base run, it is set 1. For policy test change to 2–3.
	*h*	100	scaler	For base run, it is assumed people decrease activities by exp(-1), by about 2/3, if average daily cases reach to 1% (*= 1/h*). For policy test, *h* changes to 300.
	*β*_30−60_	1	scaler	In the base run, it is assumed all faculty staff have to participate in in-person activities. In policy experiments we can change these values.
	*β*_>60_	1	scaler	
**Fatality**	*f*_<30_	0.004%	scaler	Values from CDC are used [21]. Based on age distribution of students and faculty/staff in the home institution average fatality rate of age categories are estimated.
	*f*_30−60_	0.05%	scaler	
	*f*_>60_	3%	scaler	

Our main simulation experiments include the base run simulation and four major policies, as listed in Table 3. We also test different combinations of these policies.

**Table 3:** Simulation experiments.

Tests	Operationalization
Base run	Table 1 parameter values are used.
P1: Proactive testing	*θ* = 2, *y* = 1.5, *T*_*C*_ = 1000.
P2: Mask use adoption	*M* = 80%. The value represents 40% reduction in infectivity.
P3: Risk communication	*h* = 300
P4: Remote work for high risk	*β*_>60_ = 0
Post-analysis test	The model is calibrated for Virginia Tech. Test data are input. Best estimates for the policy parameters are used as *M* = 70%, *β*_30−60_ = 0.5, and *β*_>60_ = 0.95. Using Markov Chain Mote Carlo simulation method, the joint distribution for the three unknown parameters of $a_{I_{U}}$ (we assume $a_{E}\;=\;2a_{I_{U}}$), *R*_0_ (which sets *i*_*N*_), and *h* are estimated. The university opens at August 24, and its assumed that student arrival is normally distributed around August 22 for a period of 2 weeks.

### 2.4. Model testing

The model is complex and incudes various details about university contexts and behavioral responses to the increasing cases. However, it is still a simple representation of the reality. As such, the model was carefully tested before conducting our main simulation experiments. These validation tests were intended to build confidence in the usefulness of the model. We followed various validation tests used in system dynamics modeling, such as tests of structural and behavior validity [24]. For example, we checked for unit consistency and simulation robustness in extreme conditions. Overall the model was robust.

As described later in this paper, we also conducted a post-analysis reflection where the model’s predictions were tested against how the pandemic unfolded in our home institution, by replicating the data.

## 3. Simulation results

Our simulation experiments include a base-run simulation to examine overall trends, followed by a sensitivity analysis and a range of simulation-based what-if analysis.

### 3.1. Base run simulation

Our hypothetical scenarios include a population 25,000 students and 3,000 employees (faculty and staff). The students arrive on campus in September, and it is planned that the semester consists of a 90-day period unless an early closure is announced. Early closure happens when the moving average of daily cases passes 100 individuals per day.

We first run the model for a base run scenario that includes minimal policy interventions. In this scenario, testing is reactive and based on demand from individuals, while the test capacity is relatively reasonable for reactive testing at 500 daily tests (about 1.6 test per person throughout the semester). Mask adoption is assumed to be rare in this scenario. We assume all students and employees have initially decided to attend their classes or work in-person, however, their decision may change as the daily number of confirmed cases increases. We assume that students and universities only observe confirmed cases (cases diagnosed by tests), thus the threshold for early closure is based on confirmed cases, while the actual cases might be more than this number.

**Fig 4:**
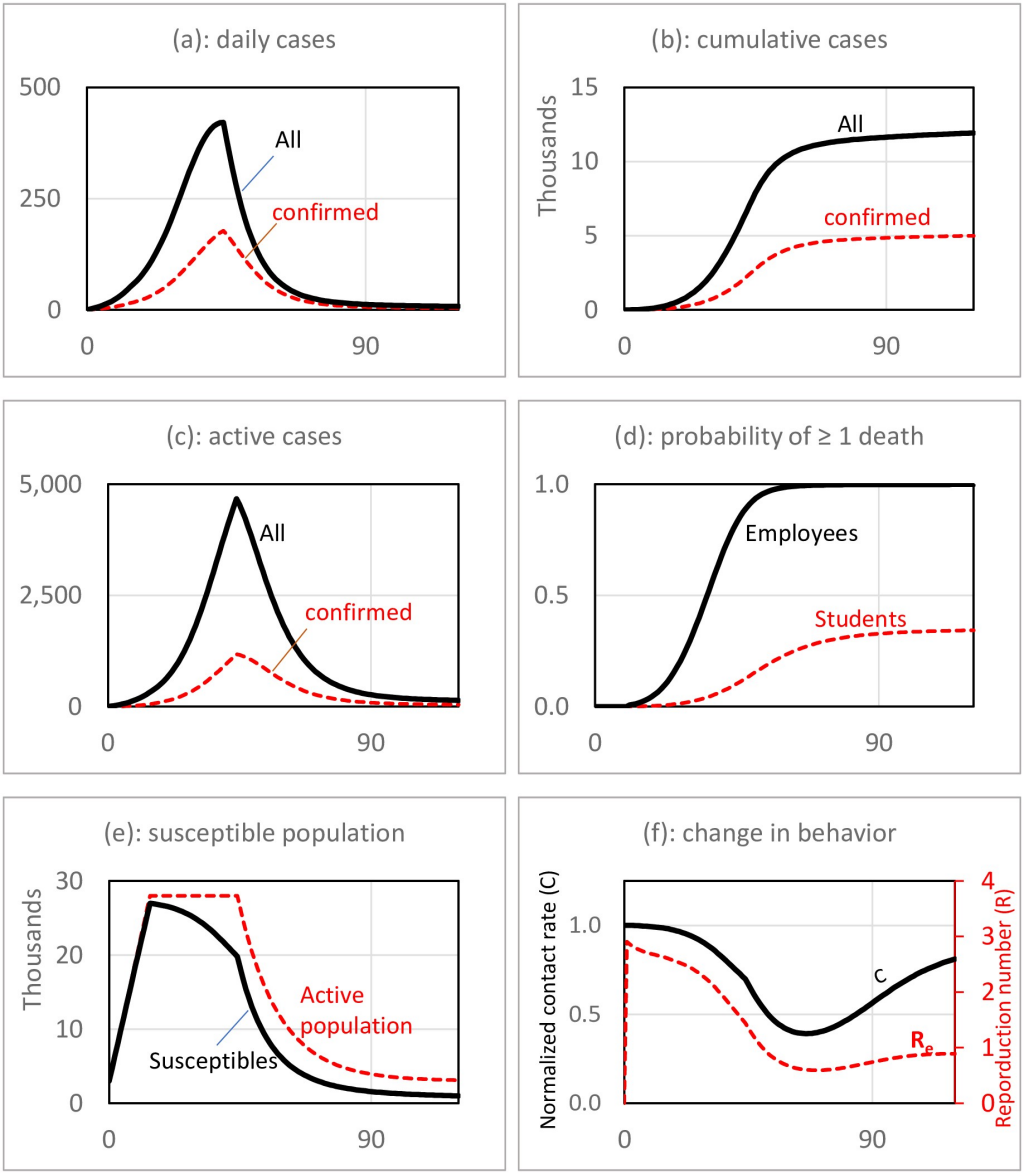
Base run simulation absent major policy interventions other than limited reactive testing. While the planned closure date is t = 90, the average of confirmed daily cases reaches the threshold for early closure at t = 43 and school closes.

Results for the base run scenario are reported in Fig 4. Based on the simulation results, the early outbreak is very likely. The number of cases start growing exponentially from early days, and it appears that the university will need to go to remote operation by the middle of the semester. Panel (a) shows that the number of confirmed cases grows fast, and by the middle of semester it passes the 100-case threshold, the assumed turning point to full remote operation. However, the actual cases are more—in our scenario it is more than twice the confirmed cases. This is due to the fact that many cases are asymptomatic and will not be diagnosed and recorded. By moving to remote operation, the number of daily cases starts declining. Panel (b) shows that the semester ends with a total of more than 11,900 cases (about 5,000 confirmed cases). Panel (c) shows the number of active cases that spread the disease on campus. Note that if the university had a higher threshold for closure and decided to operate until the end of the planned closure date of 90 days, the number of cases would have been higher.

In this scenario, the estimated probability of having a case of death is concerning. Panel (d) shows that under the base run scenario, it is very likely to have a case of death among employees who are on average older than students. The probability of having at least one death among faculty and staff reaches one by the end of the semester. For students, there is one-third of a chance of experiencing at least one case of death.

Effects of behavioral and policy feedbacks are captured in panels (e) and (f). Panel (e) depicts changes in the susceptible population. As students leave the campus after early closure, the active population declines in the town leading to a less-susceptible population. This is the main mechanism behind controlling the disease spread. Panel (f) shows how students respond to the growing number of cases by practicing social distancing. The average contact rate per individual declines by more than 50% after the peak of daily infectious cases. Public reaction helps slow the spread of the virus, but in this scenario, it is not enough to contain the outbreak, and eventually the school has to close. The change in public behavior and school closure decrease the reproduction number (R_e_), which stabilizes around one by the end of the simulation period.

### 3.2. Sensitivity analysis

Although many of the parameter values are based on the literature or from trustable sources such as the Centers for Disease Control and Prevention, they are still subject to uncertainties. Effects on the simulation outcomes of change in some of the parameters are predictable. For example, a higher infection fatality rate would result in a higher probability of death, and a higher infectivity would result in a faster growth in number of cases. Probably the most uncertain parameters are behavioral, related to people’s sensitivity to new cases and the threshold for university closure. These two parameters are specific to university contexts and prior data were not available for them. In addition, R_0_ of universities can be different from prior estimates. We conduct a sensitivity analysis for these parameters. Note that a sensitivity analysis for R_0_ can represent change in many parameters such as *α* and *μ* (relative infectivity of exposed and symptomatic to asymptomatic), infection duration, or contact rate.

Table 4 shows the effects of change in parameter values on simulation outcomes, including total number of cases, probability of death for employees and students, and whether or not the university experiences an early closure and an early outbreak (defined as having more than 1,000 cumulative cases during the first month).

**Table 4:** Results of sensitivity analysis for uncertain parameters.

	Cumulative cases	probability of ≥ 1 student death	probability of ≥ 1 employee death	Early closure*	Early outbreak**
Base run	11,900	0.34	1.00	Yes	Yes
*R*_0_					
+100% change	17,300	0.46	1.00	Yes	Yes
+50% change	14,300	0.40	1.00	Yes	Yes
-50% change	4,700	0.14	0.91	No	No
*h*					
+100% change	10,300	0.30	1.00	Yes	Yes
+50% change	10,800	0.31	1.00	Yes	Yes
-50% change	13,900	0.39	1.00	Yes	Yes
School closure threshold					
+100% change	20,300	0.51	1.00	No	Yes
+50% change	15,100	0.41	1.00	Yes	Yes
-50% change	9,100	0.27	0.99	Yes	Yes

*: closure before t = 90

**: in one month, they pass total of 1,000 cases.

The results show that the model outcomes remain qualitatively consistent. A lower basic reproduction number is one of the few parameters that can help avoid an early outbreak. If the university starts with an *R*_0_ = 1.5, the final number of cumulative cases will decline by more than 60%. Higher values of *R*_0_ results in more cases than the base run. Higher sensitivity of students to the number of cases, represented by change in *h*, can marginally help decrease cumulative cases. Finally, higher values for school closure threshold make the situation worse by expanding the duration of the semester, and adding to number of cases.

The base run scenario is not desirable due to the several reasons. In this scenario, about half of the students are infected by the virus, and the chance of experiencing a case of death is considerable especially among faculty and staff. In addition, in most cases, the university fails to operate until the end of the 90-day period and closes by the middle of the semester. While the early closure prevents virus transmission from the remaining healthy students and employees, administratively, it signals a failure. Given all the risks, it is not reasonable to even start on-campus operation. Next, we test the effects of different policies to avoid an outbreak.

### 3.3. Policy experiments

Although from the base run scenario it appears that remote operation of the university from the beginning of the semester is a reasonable policy, we use the model to test a range of alternatives and examine their effectiveness relative to the base run scenario.

A wide range of policies exist that can be tested for containing the spread of the disease. Here we focus on four major policies, and later examine the effects of combining them. The policies include:

- P1: More proactive and quick testing,
- P2: High mask use adoption,
- P3: Better risk communication with students, and
- P4: Remote work for high-risk individuals.

Under the first policy, we double the testing capacity and testing frequencies and shorten the time from test to result to 1.5 days. In the second policy, we test the effect of 80% mask adoption. The third policy targets student sensitivity and responsiveness to the number of cases. We investigate the effects of increased population sensitivity to daily cases that can lead to a quicker reaction and decline in contact rates. In this test, we triple their sensitivity to daily cases compared to the base run scenario. The last policy assumes a lower level of in-person participation for employees. In this scenario, we assume all employees over age 60 to work remotely.

**Fig 5:**
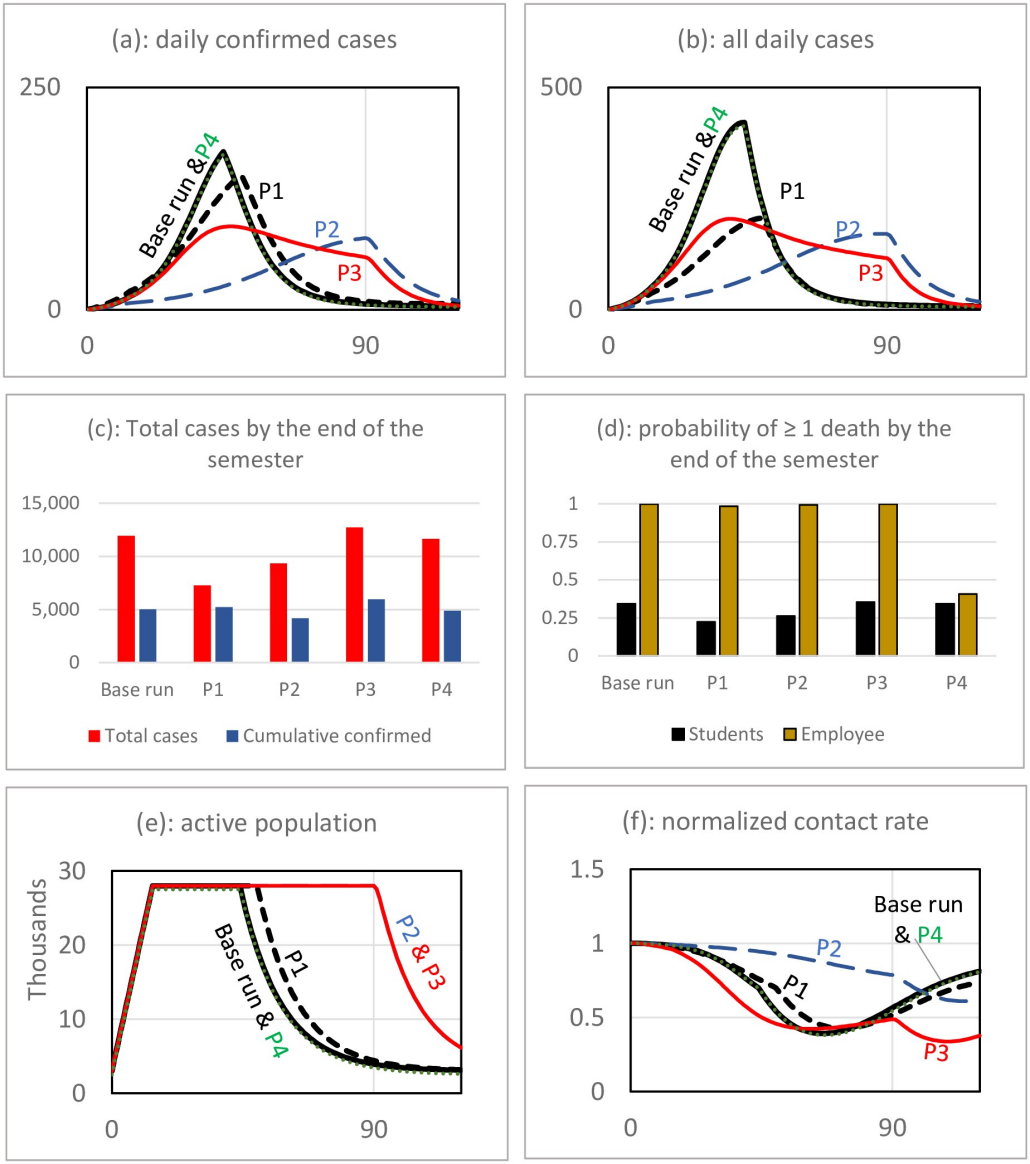
Policy experiments. Note: P1: More proactive and quick testing; P2: High mask use adoption; P3: Better risk communication with students; P4: Remote work for high-risk individuals.

Fig 5 shows the results of the policy experiments and compares them with the base run scenario. Panels (a) and (b) depict the daily cases of infection and confirmed cases and show that, in comparison to the base run, they can take different trajectories. As shown in panel (a), with the more proactive and quicker testing policy (P1), the peak in the number of daily confirmed cases slightly declines, while the actual number of daily cases, shown in panel (b), substantially decreases. This means that proactive testing results in finding a larger fraction of cases, and it helps slow down the spread of the disease. However, this result is not as obvious for policymakers and the public, whose main information source is the number of confirmed cases. Consistently, panel (c) shows that the cumulative number of cases declines considerably for P1, while the cumulative number of confirmed cases may even increase. The decline effect in actual cases on the probability of experiencing a case of death among students and employees is marginal (panel d). Since perceptions are formed based on detected cases, it is important to note that while the policy helps decrease the number of infected students, the university experiences a similar level of pressure to announce an early closure. Panel (e) shows that the university closes around the same time as the base run scenario. The effect of the policy on student contact rate is similar to the base run (panel (f)). Altogether, with more proactive testing, a higher fraction of sick individuals is found and isolated, the virus transmission slows, and the actual number of cases decline. Yet, in finding more cases, the university still faces public pressure for early closure and, absent other measures, it closes by the middle of the semester.

The second policy experiment focuses on high mask adoption at 80% (P2). Under this scenario, daily cases are flattened, which helps secure the health-sector capacity. The university ends up with about 20% percent fewer cases by the end of the semester; however, the total number of infected individuals remains large, on the order of one-third of the entire population. There is no observable effect on decreasing the likelihood of death in this scenario. Panels (e) and (f) show that under this scenario, the university is taking a different route. It can operate until the end of the pre-planned closure date of 90 days. The decline in contact rates is also smaller, due to the fact that daily cases are flattened and with mask adoption, infected contacts are leading to less transmission. In sum, mask adopton by itself flattens the curve, although the area under the curve of daily cases (i.e., cumulative cases) remains relatively large, and the likelihood of experiencing a case of death, especially among employees, is considerable. Under this scenario, the university operates until the end of the semester.

The third policy (P3) relies on more risk communication with students, assuming that with better communications, students will be more responsive to the number of cases, change their interactions dramatically, and practice social distancing protocols. The overall effect is flattening the daily cases. However, the policy loses its effectiveness through success in controlling the cases, leading to more complacency when the number of cases decline. A counterintutive result of this test is that by the end of the semester, the cumulative number of cases is larger than the base run because the school is solely relying on student reaction and, given the decline in daily cases, the university keeps operating until the end of the semester. In this scenario we observe that the cumulative number of fewer daily cases over the period of a semsester exceeds the cumulative number of higher daily cases in the base run that led to an early closure. Due to increasing the cumulative number of cases, the policy also increases the risk of having at least one case of death among students.

Finally, limiting social interactions of the high-risk population is tested in P4. Under this policy, the most vulnerable population of our model—i.e., older individuals—minimizes their contacts by completely switching to remote working. Since this population is small, the effect on the number of daily cases is negligible. Cumulative cases are in the same range as the base run, and the universities ends up closing early in the semester. However, the policy results in a significant decline in the probablity of death among employees (panel (d)). It appears that this policy only addresses one outcome measure and should be supplemented with other policies.

The analysis shows that none of these policies solely contain the spread of the virus in a university setting. Some of the options lead to flattening the curve, but this comes at the cost of continuing until the end of the pre-planned closure date, and consequently the cumulative number of cases remains high or even increase. In a sense, early closure is saving many students from the virus. The probability of having a case of death among students remains around the same range as the base run, and only the last policy helps decrease the likelihood of death among older employees. The estimated one-third probability of having one case of student death is still beyond a reasonable range for having students on the campus.

### 3.4. Combination of policies: Nonlinear effects

To shed more light on the effects of the policies, we test the effect of implementing different combinations of the policies simultaneously. The results are interesting and show non-linear incremental effects. In short, it appears that when multiple policies are simultaneously implemented, the system benefits from the synergic effects. Specifically, the effect of implementing multiple policies can be more than the sum of the effect of each individual policy. This implies that policymakers should not seek a silver bullet but rather implement all policies carefully.

**Fig 6:**
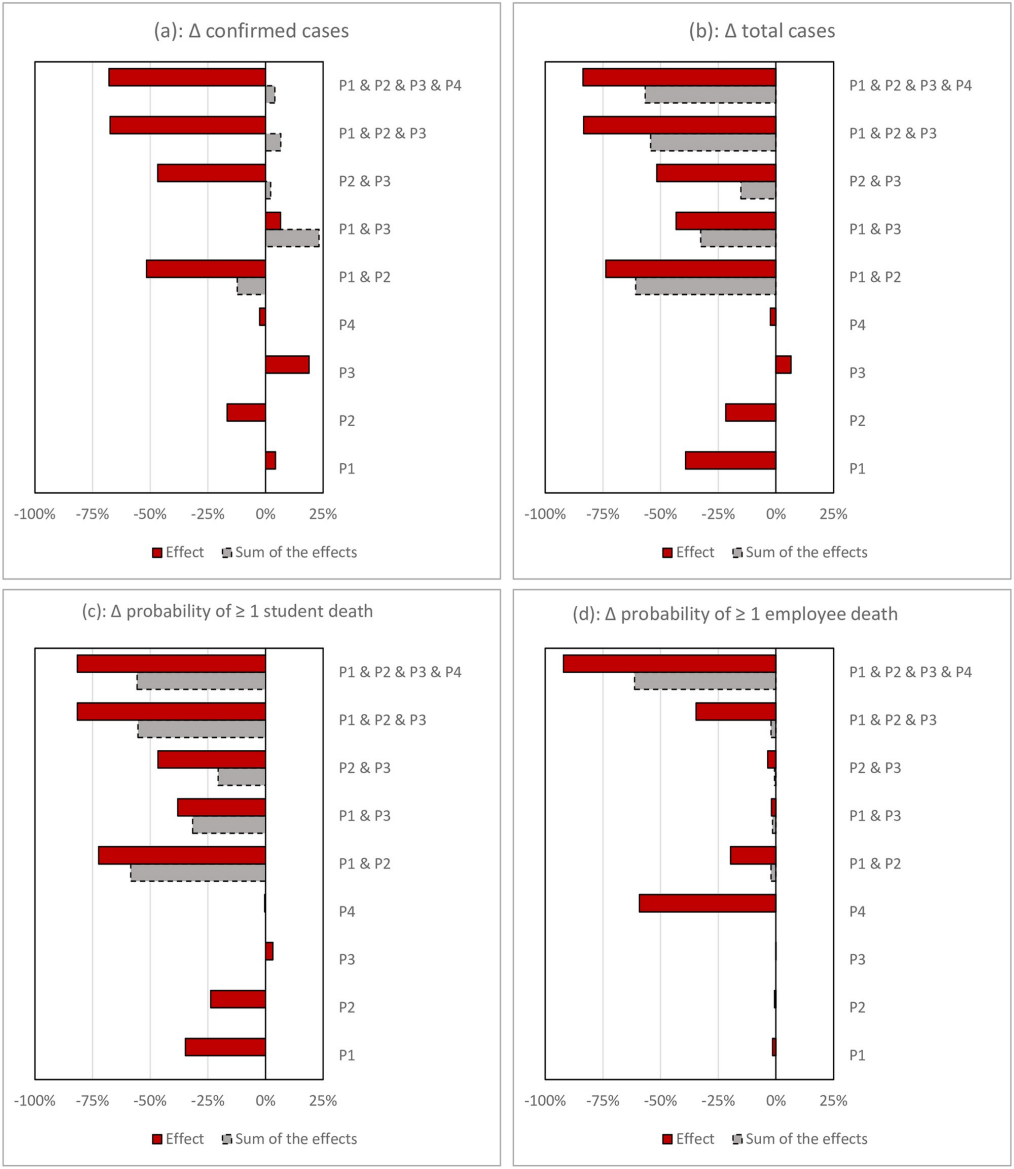
Non-linear incremental effects of policies: The effect of simultaneous implementation of multiple policies is more than sum of the effect of each policy (sum of the effects). Note: P1: More proactive and quick testing; P2: High mask use adoption; P3: Better risk communication with students; P4: Remote work for high-risk individuals.

Fig 6 depicts the non-linearity. Panel (a) shows the percentage decline in the cumulative number of confirmed case and compares the effect of P1 to P4 with the effect of simultaneously implementing some or all of them. For example, while P1 increases confirmed cases by 4% and P2 decreases confirmed cases by 17% (sum of the effects is 13%), when implemented together the effect is a 52% decline in confirmed cases. Results shown in panel (b) for the actual number of cases are also consistent. P1 and P2 separately decrease total cases by 39% and 22% (total of 61%), but when implemented simultaneously, the total cases decline by 74%. The synergy occurs because when mask-wearing is adopted, the number of cases decreases; thus the extra test capacity will be sufficient and lead to finding more asymptomatic cases, further decreasing the transmission of the disease. In simpler words, mask adoption makes proactive testing more effective. Panels (c) and (d) also show consistent results for the nonlinear effect of simultaneous implementation of the two policies on the probability of death.

A similar pattern is observable when the first and third policies of proactive testing and risk communication are performed together (P1 and P3). We noted that better risk communication substantially decreases the peak in the number of cases. This can make proactive testing more effective during the early periods and lead to a further decline in the number of cases (Panel b). The number of confirmed cases still increases compared with the base run, as both of the policies were contributing to more confirmed cases.

The combination of mask adoption and risk communication (P2 and P3) is also effective in substantially decreasing the number of cases. It is interesting to note that while a sole implementation of risk communication was mainly shifting the burden onto students and resulting in more cumulative cases, with mask adoption the direction of the effect changes and we see a decline in the number of cases. Risk communication mainly stabilizes the number of cases particularly after the early predicted outbreak, and mask adoption helps mitigate the chance of an early outbreak by starting with a lower reproduction number.

More interesting results appear when P1, P2, and P3 are implemented together. The result is a 67% decline in confirmed cases and an 83% decline in total cases. This is much more than the total of the effect of solely implementing each of the policies as shown in panels (a) and (b). The policies when implemented simultaneously decrease the likelihood of a student death by about 82% (panel (c)). When we add P4, keeping high-risk individuals working remotely, the probability of death for employees also declines by 92% (panel (d)). Note that while P4 was very effective in decreasing the likelihood of death among employees, the effect of sole implementation of P4 was on the order of 70%, and the sum of the effects on an employee death of all policies is 74%—both much lower than the 92% that we gain when we simultaneously implement all polices. The reason is related to the synergetic effect of implementing all policies together on total cases: the fewer number of cases lead to a smaller likelihood of death too.

**Fig 7:**
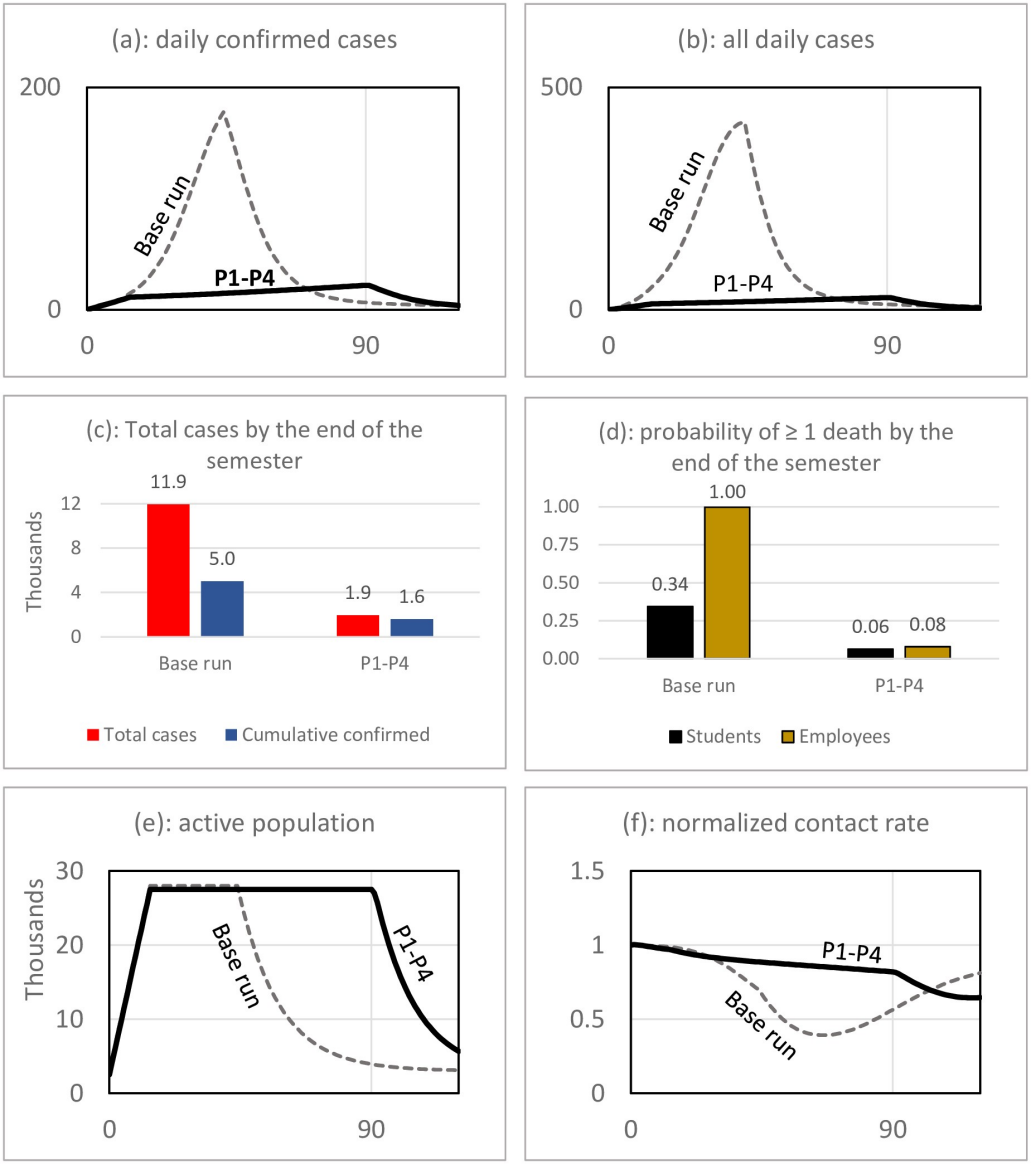
Outcome measures for implementing all policies simultaneously in comparison to the base run scenario.

Fig 7 depicts graphs over time and compares the scenario of all four policies with the base run. It shows that this scenario can result in controlling the outbreak, ending with a total of 1,900 cases (about 1,600 confirmed). Overall, it appears that unless all policies are implemented together—with the assumed parameters for covid-19—an outbreak is very likely. We should also consider that the described policies come with major implementation challenges.

Altogether, the analysis reveals that (a) an early outbreak is likely, and it is safer to move to full remote operation from the beginning of the semester without students coming to the campus, and (b) if decided otherwise, all policies should be carefully implemented to mitigate the potential consequences.

## 4. Post-analysis reflections

This research was primarily conducted during the summer of 2020 to inform university decision-makers regarding the upcoming fall semester. Ultimately, universities’ decisions were mixed. While places such as Harvard University decided early on to mainly focus on remote operations and avoid on-campus activities, many universities, including our home institution opened their campuses, and invited students for in-person or hybrid classes. In a few weeks, however, several universities that had opened their campuses, such as the University of Notre Dame, the University of North Carolina at Chapel Hill, and Michigan State University ended up switching to remote operations after experiencing about 100 cases of covid-19 [25, 26]. Many other universities tried to control the outbreak by implementing more restrictive actions such as suspension of students who violated social distancing protocols.

As of December 20, 2020, it is estimated that about 400,000 university students have been infected by SARS-CoV-2 virus [27]. The actual numbers can be much higher due to asymptomatic cases. One of the possible negative consequences of opening universities has been an increase in deaths at nursing homes in college towns [28]. On December 12th, *The New York Times* reported that “deaths from the coronavirus have doubled in counties with a large college population, compared with a 58 percent increase in the rest of the nation” and most of the victims have been “older people and others living and working in the community” [28].

**Fig 8:**
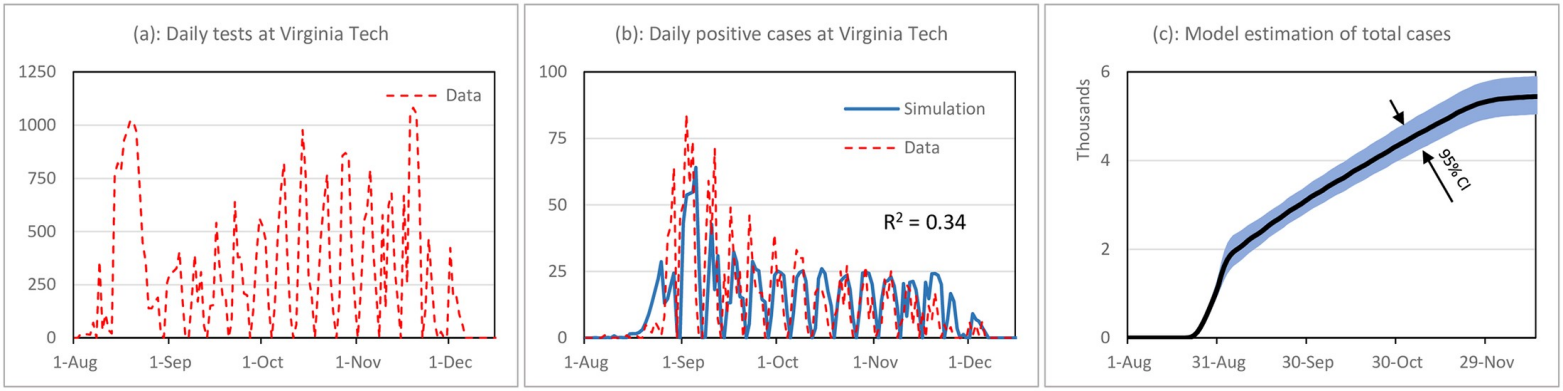
Replication of data from Virginia Tech and estimation of parameters.

As a post-analysis reflection, our model can be tested against the data. The model can be also used to estimate some of the unknown variables such as undocumented cases of the disease. To that end, we use data from our home institution and test the model’s fidelity in replicating the data and its estimation of unknown parameters conditional on the observed data. The data and simulation results are shown in Fig 8. Since the number of daily tests changes over the semester and declines during most of the weekends (panel (a)), we enter the daily test data directly as an input to the model and let the model produce the number of daily cases over time. The procedure is explained in Table 3, and the model in Vensim is provided. For calibration, we form a likelihood function for observing daily confirmed cases conditional on model parameters. Conducting a Markov Chain Monte Carlo simulation, the joint posterior distribution of the model parameters is estimated.

Panel (b) of Fig 8 shows that simulation results closely follow the historical trends (R^2^ = 0.34). The model’s estimation for the total number of cases including off-campus residents and undiagnosed cases is reported in panels (c) with a total of 5,474 (95% CI: 5070–5939).

The model estimates that about 1.2% (95% CI: 0.6%-1.8%) of arriving students were infected at time of arrival ($a_{E}+a_{I_{U}}$). Interestingly, the model estimates a much larger value for *R*_*0*_ than our initial assumption in the hypothetical case, at 8.4 (95% CI: 6.4–10.7) which might be due to the fact that students overall have a much higher contact rate than average individuals, particularly early on in the semester when they attend more social gatherings. Sensitivity to daily cases, *h*, is estimated at 4,736 (95% CI: 4,070–5,416), which shows that students and employees quickly responded to daily cases, and the system reached the long-term equilibrium of *R*_*t*_ ≈ 1 at an average daily case of 16 person per day. This depicts a very high sensitivity to daily cases possibly coming from frequent communications of the administration with the students and employees.

## 5. Model files

A web app is developed for what-if analysis. Users can change different parameters and examine simulation results. The model in Vensim software is also provided as a S1 File. After installing Vensim PLE (free), the model can be used for what-if analysis. In addition, a model calibrated to Virginia Tech data is provided in the S1 File. This model needs to read Virginia Tech data (provided), and will work with Vensim DSS.

## 6. Conclusion

In this paper, a simulation model of the spread of covid-19 at a hypothetical university is reported, and the model is used to estimate effects of different policy measures on containing the spread. The model focused on the dynamics of the system [29] as affected by behavioral feedback loops [30], including human reactions to the growing number of cases and the administration’s decision for early closure. The main contribution of this work is to offer insights into the relative effects of different policies to contain an outbreak in a university setting. We also offer a platform for a what-if analysis so universities can use the platform to test different policies. Our results reveal several sources of complexity in this system and show how universities are susceptible to an outbreak even when different policies are implemented. In this paper, attention should be paid to relative changes (qualitatively) in outcome variables rather than exact quantitative numbers. Furthermore, optimal policies for different universities may differ based on their population, age distribution, and initial cases of infection.

As a proof-of-concept, we simulated the model and analyzed the results for a hypothetical university of 25,000 students and 3,000 faculty/staff members more likely to be located in a college town with close student interactions. Parameter values were selected in a way that makes this proof-of-concept simulation close to conditions that many U.S. universities are experiencing.

Our results corroborate with other modeling studies that have cautioned universities about having on-campus students during the fall 2020 semester (For a summary, see [31]). Overall, our simulation results show that within reasonable ranges of parameter values, the disease is likely to spread very fast, and to avoid catastrophic outcomes, early closure is unavoidable for many universities. In-person classes come with a considerable risk and the administration should pay close attention to various indicators for early closure. In the long run, students and faculty react to the outbreak, and even if the universities decide to continue their operations, classes are likely to result in very low in-person attendance. However, this means that by the end of the semester, many students and possibly faculty and staff will be infected. Given the disease’s unknown log-term effects it appears that it is not worth the risk to open the campuses.

In cases that universities decide to (partially) hold in-person classes, several policies should be carefully implemented. The model shows how the system is fragile and how multiple policies are needed to see a decline in both the number of cases and the probability of death. We show that there is no silver bullet, and to control the spread of the disease, attention should be paid to a portfolio of actions that includes proactive quality testing, minimum delay between symptom onset and test results, enforcing mask use, and social distancing. Higher testing frequencies might require the use of techniques that scale up testing with a limited extra burden on staff [32, 33]. In addition, working remotely should be strongly suggested to the high-risk population of over 60 and the majority of individuals between 30 and 60. Only when all policies are in place can we expect desirable outcomes. However, even in the most favorable conditions, the model estimates a relatively considerable probability of death in a period of 90 days, and thus careful attention should be paid to different indicators such as average daily cases and active cases, and closing earlier when the trends appear alarming.

Our simulation results for combining different testing policies show a synergetic effect between policy interventions [34]. Sole implementation of some of the policies may affect results with negative consequences. For example, only relying on risk communication flattens the curve of daily cases by shifting the burden onto students rather than announcing an early closure, which can potentially lead to a higher number of total cases. However, when risk communication is implemented with more frequent and rapid testing, it improves people’s responsiveness and substantially decreases cases. Such non-linear effects are common characteristics of complex systems. The implication is that a close monitoring of the system and implementing all policies are required.

Our study has several limitations. We did not consider complexities associated with healthcare delivery, such as limited hospital capacity and emergency rooms [35]. An outbreak in a college town that is unprepared for a large number of patients can put an extra burden on the healthcare system, affecting services and recovery time. Details of student interactions with town residents can be further modeled. Social network heterogeneities can affect the rate at which the disease spreads. These factors were neglected or simplified in our analysis to keep the model simple and focus on some of the important behavioral feedback structures.

Furthermore, our main analysis was based on a hypothetical case to demonstrate first-order insights from the model. Model calibration plays a crucial role in improving projections and estimating cases of documented and undocumented infection [7, 36], and this model can benefit from the same practice. The exact magnitude of effects of mask enforcement, social distancing policies, and student compliance can be later estimated by statistical methods [37] and parameter values can be further updated. As a post-analysis reflection, we showed the model can replicate data from our home institution and estimate the total number of cases. In future studies, the model can be tested against a larger sample of universities.

## Supporting information

S1 File Model and data. The zipped folder includes the simulation models in Vensim (COVID-Universities-V10-generic.mdl and COVID-Universities-V10c-data input.mdl), the data file and other inputs to the simulation model, and a short instructional document about running the model.
(ZIP)
