# Supplementary material for: Simulation-based what-if analysis for controlling the spread of Covid-19 in universities
Source: PLoS One. 2021 Feb 1;16(2):e0246323. doi: 10.1371/journal.pone.0246323 (PMC7850497; doi:10.1371/journal.pone.0246323)
Supplement: S1 File — The zipped folder includes the simulation models in Vensim (COVID-Universities-V10-generic.mdl and COVID-Universities-V10c-data input.mdl), the data file and other inputs to the simulation model, and a short instructional document about running the model. (ZIP) [file pone.0246323.s001.zip › Model/Read me.pdf]

Instruction for supplementary material of

Simulation-based What-if Analysis for Controlling the Spread of Covid-19 in Universities

Navid Ghaffarzadegan (navidg@vt.edu)

Department of Industrial and Systems Engineering, Virginia Tech

1. A web app is provided for what-if analysis. Users can change different parameters and examine simulation results with no need to install any software.

2. The model in Vensim software is provided as a supplementary. Install Vensim PLE (free) from https://vensim.com/free-download/. Then open the file “COVID-Universities-V10-generic.mdl”. You will see the model’s dashboard. You can run the model in the “SyntheSim mode” by clicking on the icon (find the green button about the top of your screen, after “Simulate”). In this mode you change the parameters and see simulation results in the real time.

   Using PgUp and PgDn on your keyboard, you can go to different layers of the model. Continue PgUp, until you stop at the view “Infection dynamics” where you see the basic SEIR structure. With Vensim PLE you will see all the variables and parameters. If you are using Vensim DSS, using arrow keys on your keyboard you can unfold the model and explore different variables.

   You can explore the equations, by first stopping the simulation run, clicking on the icon “equations” (note the red button *f(x)* at the top of your screen) and then on each variable.

3. In addition, a version of the model calibrated to Virginia Tech data is provided (COVID-Universities-V10c-data input.mdl). This model needs to read Virginia Tech data (provided), and will work with Vensim DSS. Optimal parameter values are in MCMC.out, and the data are available in the excel sheet and the vdfx file. Vensim DSS will automatically read them as long as they are kept in the same folder.
